# Dermoid Cysts of the Asterion: An Unusual Location for Unusual Dermoids, Radiological Findings and Neurosurgical Implications

**DOI:** 10.3390/tomography8020093

**Published:** 2022-04-14

**Authors:** Alessia Guarnera, Guido Trasimeni, Andrea Romano, Antonella Stoppacciaro, Mattia Serio, Massimo Miscusi, Alessandro Bozzao

**Affiliations:** 1 Department of Neuroradiology, S. Andrea Hospital, University Sapienza, 5, 00185 Rome, Italy; guido.trasimeni@uniroma1.it (G.T.); andrea.romano@uniroma1.it (A.R.); mattia.serio@uniroma1.it (M.S.); alessandro.bozzao@uniroma1.it (A.B.); 2 NESMOS Department, S. Andrea Hospital, University Sapienza, 5, 00185 Rome, Italy; massimo.miscusi@uniroma1.it; 3 Surgical Pathology Units, Department of Clinical and Molecular Medicine, Sant’ Andrea Hospital, La Sapienza University, 5, 00185 Rome, Italy; antonella.stoppacciaro@uniroma1.it; 4 Department of Neurosurgery, S. Andrea Hospital, University Sapienza, 5, 00185 Rome, Italy

**Keywords:** asterion, dermoid cyst, lateral cranial base approach, computed tomography, magnetic resonance imaging

## Abstract

Asterion is an uncommon site for lesions, especially dermoid cysts. We report a case series of three asterional intracranial dermoid cysts, which, to the best of our knowledge, have never been described before. Patients presented with non-specific symptoms and underwent surgical excision of the lesions. It is crucial to correctly diagnose intracranial masses and to identify their relationships with surrounding anatomical structures, especially if the location is unusual as the asterion, to plan surgery. The challenge of this tumor location is to preserve the venous drainage system during surgical procedures, because of the contiguity between the asterion and the transverse–sigmoid junction. Rupturing or damaging of the venous drainage system have been proven to be catastrophic because they lengthen surgical time and present dire consequences for patients. In conclusion, it is crucial to familiarize with atypical dermoid presentation to ensure proper diagnoses and to perform adequate imaging for optimal surgical planning.

## 1. Introduction

Asterion derives from the Greek word “aster”, meaning star, and it indeed represents a star-like junction of the parietomastoid, lambdoid and occipitomastoid sutures. It is a useful landmark for the transverse–sigmoid sinus junction [1,2] and for lateral cranial base surgical approaches [3]. Few studies have reported the asterional location of intracranial masses, and only one study has reported an asterional extracranial dermoid cyst [4]. Dermoid cysts are rare benign tumors, accounting for fewer than 0.5% of intracranial masses, and are mostly seen along the midline [5]. We report a case series of three asterional intracranial dermoid cysts: their clinical histories, their radiological patterns, and their surgical treatment.

## 2. Case Presentation

**Figure 1:**
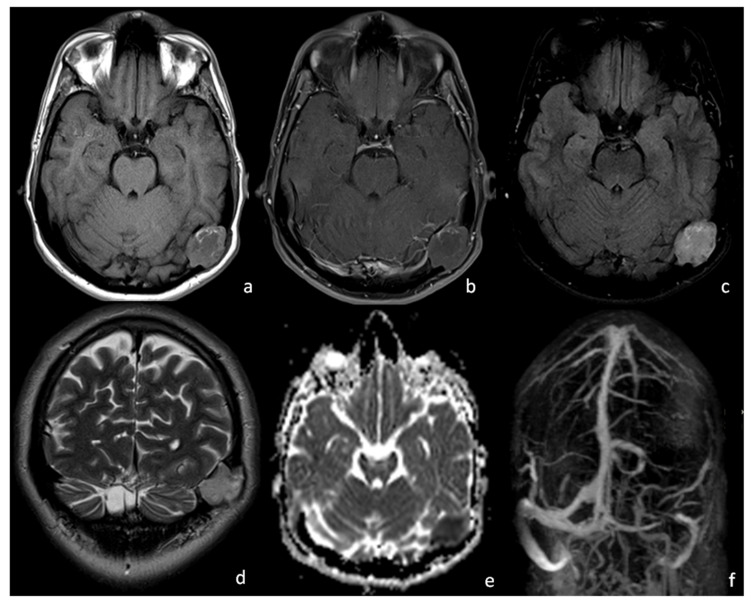
Case 1, 1.5 T MRI: on T1WI (**a**) there is evidence of a predominantly isointense left asterional mass with a surrounding, subtle, hyperintense rim; no signal enhancement is noted after gadolinium injection (**b**). The lesion appears to be unevenly hyperintense on FLAIR (**c**) and T2WI (**d**), with restricted signal on ADC (**e**). The 3D MIP reconstruction of contrast-enhanced MR venography (**f**) shows a severe stenosis of the left transverse–sigmoid sinus junction caused by the asterional dermoid cyst.

First Case: C.G., a 27-year-old man, presenting with nausea and persistent left migraine, underwent an MRI scan (Figure 1). Physical and neurological examinations and blood tests were negative. No masses were palpable. The MRI scan showed a left-sided, intracranial, and extra-axial lesion in the temporo–occipital region with a thecal epicentrum. It appeared to be isointense on T1WI and slightly hyperintense on T2WI/FLAIR with a T1WI, T2WI, FLAIR hyperintense peripheral rim (Figure 1a,c,d). The core of the lesion showed a modest diffusion restriction with no contrast enhancement (Figure 1e). The mass seemed to determine a stenosis of the left transverse–sigmoid sinus junction on the contrast-enhanced MR venography and the related 3D MIP reconstructions (Figure 1b,f). The patient underwent open surgery and had the lesion removed. Sigmoid and transverse venous sinuses were apparent and were preserved during surgery. Histological analysis confirmed the diagnosis of a dermoid cyst. Post-surgery recovery went smoothly, and the patient reported a significant improvement in his symptoms.

**Figure 2:**
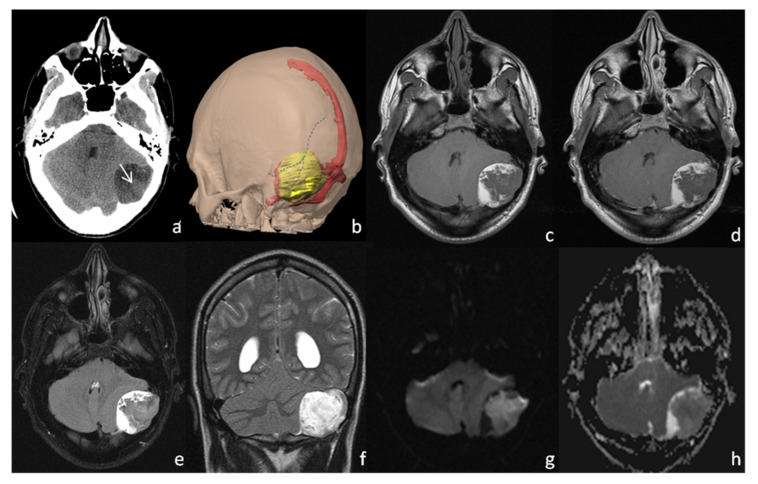
Case 2: CT scan (**a**) showing a hypodense left asterional lesion with a central, slight hyperdense area (**a**, white arrow), corresponding to an area of diffusion restriction on DWI/ADC (**g**,**h**). A 3D pre-surgical reconstruction model (**b**) was performed to correctly identify the relationships among the asterion (green dot), which is the intersection of parietomastoid, lambdoid and occipitomastoid sutures (blue dotted lines), the dermoid cyst (yellow) and the adjacent venous system (red). The 1.5 T MRI (**c**–**h**) showing a prevalently hypointense left asterional mass, with no contrast enhancement after gadolinium administration (**d**). A similar hyperintense rim is noted on FLAIR (**e**) and T2WI (**f**), whereas the mass appears to be more hyperintense on T2WI than on FLAIR.

Second Case: C.R., a 29-year-old man, presenting with persistent headache and dizziness since childhood, underwent a CT scan during an emergency health care procedure (Figure 2a). Blood test, physical, and neurological examinations were negative, aside from a mild left ear hypoacusis. No skull masses were palpable. CT scan showed a left-sided, inhomogeneously hypodense mass centered on the asterion, causing bone remodeling and two focal areas of bone erosion. For better surgical planning, a 3D model was obtained (Figure 2b). On the MRI scan, the lesion showed a T1WI and FLAIR iso-hypointense and T2WI hyperintense core, surrounded by a T1WI and FLAIR hyperintense peripheral rim (Figure 2c,e,f). The core showed a modest diffusion restriction, but no contrast enhancement (Figure 2d,g,h). The patient underwent open surgery and had the lesion removed. Histological analysis confirmed the specimen to be a dermoid cyst and its location to be extradural. During post-surgery recovery, a local infectious complication occurred, which was treated with antibiotics. After clinical remission, the patient only reported the persistence of left hypoacusis.

**Figure 3:**
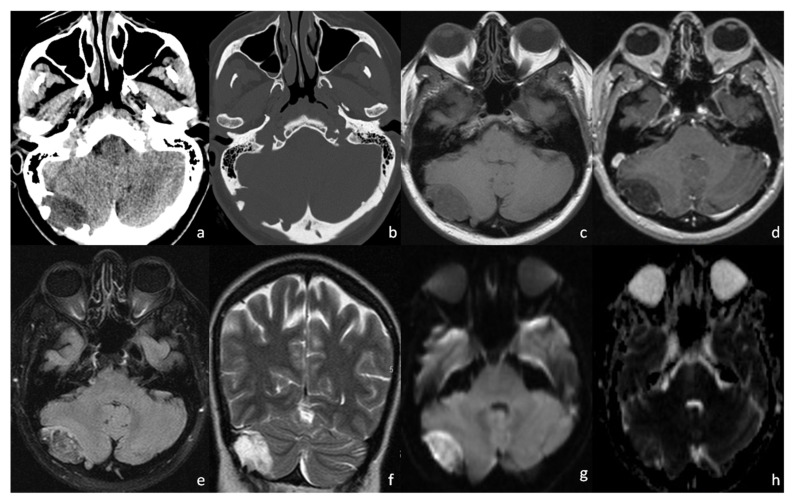
Case 3: CT scan (**a**,**b**) showing a thecal-centered, inhomogenously hypodense mass causing bone remodeling and focal areas of bone erosion. The 1,5 T MRI (**c**–**h**) of the lesion, which appears unevenly hypointense on T1WI (**c**), and hyperintense on T2WI (**f**). There is incomplete fat saturation on FLAIR (**e**) and on post-contrast 3D T1 MPRAGE (**d**); and poor restricted diffusion on DWI/ADC (**g**,**h**). On 3D T1 MPRAGE (**d**) subtle contrast enhancement of the capsule and some thin internal septa may be appreciated.

**Figure 4:**
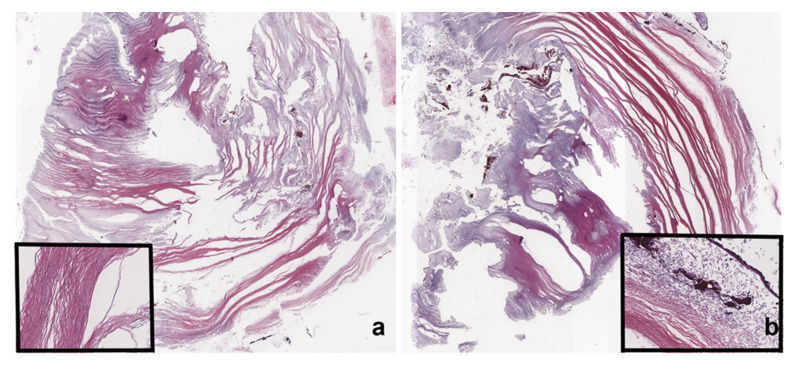
Case 3: the intracranial cyst was composed of squamous epithelia. In panels **a** and **b**, the sections show the lamellar keratin organization (highlighted in the insert in (**a**), keratin debris, and the absence of viable epithelium lining the thin wall of the cyst (insert in (**b**): wall inked by black China ink). Hematoxylin and eosin; (**a**,**b**) original magnification 1×; inserts in (**a**,**b**) original magnification 200×.

Third Case: C.M., a 52-year-old woman, presenting with headache and vertigo at first aid, underwent a CT scan (Figure 3a,b). Blood test, physical, and neurological examinations were negative, and no skull masses were palpable. CT scan showed a right-sided, hypodense mass with a thecal epicenter, causing bone remodeling and multiple areas of bone erosion (Figure 3a,b). An MRI scan showed an inhomogeneous T1WI hypointense and T2WI hyperintense oval mass (Figure 3c,f), with an incomplete fat saturation (Figure 3d,e) and poor restricted diffusion on DWI/ADC (Figure 3g,h). Contrast enhancement was subtle and limited to the capsule and some thin internal septa (Figure 3d). The ipsilateral transverse sinus was minimally compressed, but obvious. The patient underwent open surgery, and the lesion was easily removed because of its extradural location. Histological analysis confirmed the diagnosis of a dermoid cyst. In particular, the intracranial cyst was composed of squamous epithelia with a typical lamellar organization of keratin coexisting with keratin debris (Figure 4a,b). Post-surgery recovery went smoothly, and the patient reported an improvement in her symptoms.

## 3. Discussion

Dermoid cysts are rare benign tumors which account for fewer than 0.5% of intracranial masses and originate from the ectopic inclusion of epithelial cells during closure of the neural tube in the 3rd to 5th week of embryonic development [5,6].

They represent thick-walled unilocular cysts lined with a stratified squamous epithelium and contain sebaceous materials, keratin debris, and skin adnexa [5].

Intracranial dermoid cysts are presumed to arise from precocious embryological accidents, and this is the reason why dermoid cysts are almost constantly located along the midline [7], the anterior fontanel being the most common site [4,8] and the asterional region being extremely rare.

We found only one study [4] describing an extracranial asterional dermoid cyst, although the cases we report presented dermoid cysts located intracranially and extradurally.

Dermoid and epidermoid cysts may also arise from traumatic implantation [7]. We exclude this latter theory because our patients did not report any previous head surgery or trauma.

We speculate that the unusual location might be explained by delay in the closure of the neural tube at the asterion level, resulting in the inclusion of the ectoderm and subsequent formation of dermoid cysts.

Dermoid cysts may affect patients’ quality of life and may cause significant morbidity and mortality in cases of rupture [9]. Therefore, it is crucial to correctly diagnose the masses and their location, especially if the location is unusual, such as the asterion, in order to plan surgery.

CT helps to ascertain the position of the asterion and secondary bone tissue remodeling. Dermoid cysts are generally hypodense on CT scans due to their high fat content and do not exhibit enhancement after contrast administration. It is highly recommended to perform a 3D CT pre-surgical reconstruction model [10] to identify the relationships among the asterion, which is the landmark for neurosurgical access, the dermoid cyst, and the adjacent venous system.

MRIs provide crucial information to diagnose dermoid cysts and to show their relationships with surrounding anatomical structures. Dermoid cysts are generally hyperintense on T1WI and hypointense to hyperintense on T2WI, depending on the heterogeneity of the lesion. Fat suppression and diffusion-weighted sequences are recommended to confirm the diagnosis [5,11].

The first and the third cases analyzed atypical dermoid cysts, appearing to be isointense and hypointense on T1WI and slightly hyperintense on T2WI/FLAIR, with poor fat suppression and subtle diffusion restriction. Atypical dermoid cysts have seldom been described in the literature, and their unusual features have been correlated with high liquor content, minimal amounts of lipids, lipid, and keratinized debris saponification with secondary microcalcification in suspension, partially liquefied cholesterol, high protein content, and hemosiderin or iron–calcium complexes relating to previous episodes of hemorrhage within the cyst [12,13,14]. Epidermoid and dermoid cysts pathologically differ in relation to the presence of skin appendages in the cyst wall, which are present in the dermoid. If a dermoid cystic wall presents few skin appendages, the lesion may radiologically appear as an epidermoid cyst, and change in radiological appearance in time, secondary to gland secretion, to resemble a dermoid cyst [15]. Two of our lesions seemed to present features of both the epidermoid and dermoid, corroborating the hypothesis of a radiological degeneration of the epidermoid into the dermoid [15] to justify their intermediate and peculiar characteristics.

The therapy approach for these lesions is resection. The assessment of internal cranial anatomy based on external landmarks is pivotal to avoid injury to important structures during posterior cranial fossa surgery [2]. When approaching the posterior fossa through transmastoid and retrosigmoid approach [10], or through a combined petrosal approach [3], the asterion has proven to be a useful and reliable surgical landmark. In particular, burr holes at the asterion may be placed directly over the sinus, leading to potential damage [1], whereas burr holes drilled below and medially to the asterion may expose the posterior fossa with the least risk [2,16]. Moreover, the lateral suboccipital retrosigmoid approach is a standard procedure for lesions such as cerebellopontine angle tumors and for performing microvascular decompression [10]. To minimize complications, such as anatomical structures injuries and bone loss, it is paramount to expose the margin of the transverse and sigmoid sinus by making a keyhole on the transverse–sigmoid junction lateral to the asterion by a half diameter of the burr hole [10].

For these reasons, the relationship between the venous drainage system and an asterional mass should always be investigated because dermoid-encased vessels and sinuses have a high risk of rupture [5], and the position of the asterion is mostly located superficially to the transverse–sigmoid junction [2,3], which could be damaged during surgical access. The risk is higher in case of intradural masses. Rupturing or damaging of the venous drainage system during surgery has been proven to be catastrophic, because it lengthens the surgical time and represents dire consequences for patients [17,18]. Therefore, it is highly recommended to perform a 3D CT pre-surgical reconstruction model for the evaluation of the asterion [10], a contrast-enhanced MR venography and the related 3D MIP reconstructions to evaluate venous drainage.

## 4. Conclusions

It is crucial to further the understanding of atypical dermoid presentation to ensure proper diagnoses and to perform adequate CT and MR imaging for optimal surgical planning.

## Data Availability

The data are available from the corresponding author, A.G., upon reasonable request.
